# Supplementary material for: Assessment of Ambient Air Toxics and Wood Smoke Pollution among Communities in Sacramento County
Source: Int J Environ Res Public Health. 2020 Feb 8;17(3):1080. doi: 10.3390/ijerph17031080 (PMC7037835; doi:10.3390/ijerph17031080)
Supplement: Supplementary file 1 [file ijerph-17-01080-s001.pdf]

International Journal of
Environmental Research and Public Health

MDPI

Supplemental Materials

Supplemental Tables

Table S1. Summary of Kruskal-Wallis estimates of distribution differences between daytime and nighttime concentrations of HAPs. All values in **bold** are statistically significantly higher daytime concentrations at greater than 95% confidence level. Values in italics are daytime concentrations at 90% confidence levels.

Pollutant	64th Street	ARB T Street	Darwin	DPM	Socorro	Tristan
1,3-Butadiene	*0.056*	0.734	**0.023**	**0.001**	**0.002**	**0.015**
2,2,4-Trimethylpentane	*0.074*	0.395	**0.003**	**0.002**	**0.003**	**0.028**
Acetonitrile	0.854	0.308	**0.001**	**0.000**	**0.009**	0.225
Acetylene	**0.016**	0.734	**0.003**	**0.001**	**0.002**	0.157
Acrolein	0.442	**0.041**	0.954	**0.002**	0.453	0.603
Benzene	*0.074*	0.734	**0.002**	**0.001**	**0.006**	**0.028**
Carbon Tetrachloride	0.579	0.865	0.118	0.927	0.248	*0.073*
Ethylbenzene	*0.090*	0.734	**0.003**	**0.005**	**0.006**	**0.038**
m,p-Xylene	*0.097*	0.610	**0.007**	**0.005**	**0.007**	**0.021**
Toluene	*0.074*	0.610	**0.002**	**0.004**	**0.006**	**0.043**

Table S2. Significance of posthoc Nemenyi-tests for pairwise multiple comparisons for BC between each community. Values below 0.05 (**bold**) indicate a statistically significant difference between the pair of communities. The median, mean, and standard deviation (SD) of the BC values measured at each community is also indicated.

BC		64th Street	Tristan	Del Paso Manor	Darwin	T Street
	Median (Mean/SD)	0.45 (0.87/1.07)	0.40 (0.75/0.92)	0.57 (1.22/1.67)	0.45 (1.02/1.42)	0.38 (0.69/0.83)
Tristan	0.40 (0.75/0.92)	0.223	-	-	-	-
Del Paso Manor	0.57 (1.22/1.67)	**0.000**	**0.000**	-	-	-
Darwin	0.45 (1.02/1.42)	0.994	0.534	**0.000**	-	-
T Street	0.38 (0.69/0.83)	**0.069**	0.997	**0.000**	0.241	-
Socorro	0.47 (1.17/1.71)	0.922	**0.011**	**0.007**	0.615	**0.001**

Table S3. Significance of posthoc Nemenyi-tests for pairwise multiple comparisons between each community for BC_{wb}. Values below 0.05 (**bold**) indicate a statistically significant difference between the pair of communities. The median, mean, and SD of the BC_{wb} concentration measured at each community is also indicated.

**BC_{wb}**		**64th**	**Tristan**	**Del Paso Manor**	**Darwin**	**T Street**
	Median (Mean/SD)	0.15 (0.40/0.70)	0.12 (0.27/0.43)	0.26 (0.77/1.34)	0.16 (0.51/0.90)	0.14 (0.27/0.39)
Tristan	0.12 (0.27/0.43)	**0.001**	-	-	-	-
Del Paso Manor	0.26 (0.77/1.34)	**0.000**	**0.000**	-	-	-
Darwin	0.16 (0.51/0.90)	0.963	**0.000**	**0.000**	-	-
T Street	0.14 (0.27/0.39)	0.529	0.313	**0.000**	**0.096**	-
Socorro	0.14 (0.44/0.75)	0.794	0.103	**0.000**	0.246	0.998

Table 4. Significance of posthoc Nemenyi-tests for pairwise multiple comparisons between each community for BC_{ff}. Values below 0.05 (**bold**) indicate a statistically significant difference between the pair of communities. The median, mean, and SD of the BC_{ff} concentrations measured at each community is also indicated.

**BC_{ff}**		**64th**	**Tristan**	**Del Paso Manor**	**Darwin**	**T Street**
	Median (Mean/SD)	0.28 (0.47/0.28)	0.28 (0.48/0.28)	0.25 (0.45/0.25)	0.26 (0.50/0.26)	0.23 (0.41/0.23)
Tristan	0.28 (0.48/0.28)	0.833	-	-	-	-
Del Paso Manor	0.25 (0.45/0.25)	0.915	1.000	-	-	-
Darwin	0.26 (0.50/0.26)	0.341	0.979	0.927	-	-
T Street	0.23 (0.41/0.23)	0.139	0.846	0.706	0.997	-
Socorro	0.32 (0.73/0.32)	0.123	**0.002**	**0.003**	**0.000**	**0.000**

Supplemental Figures

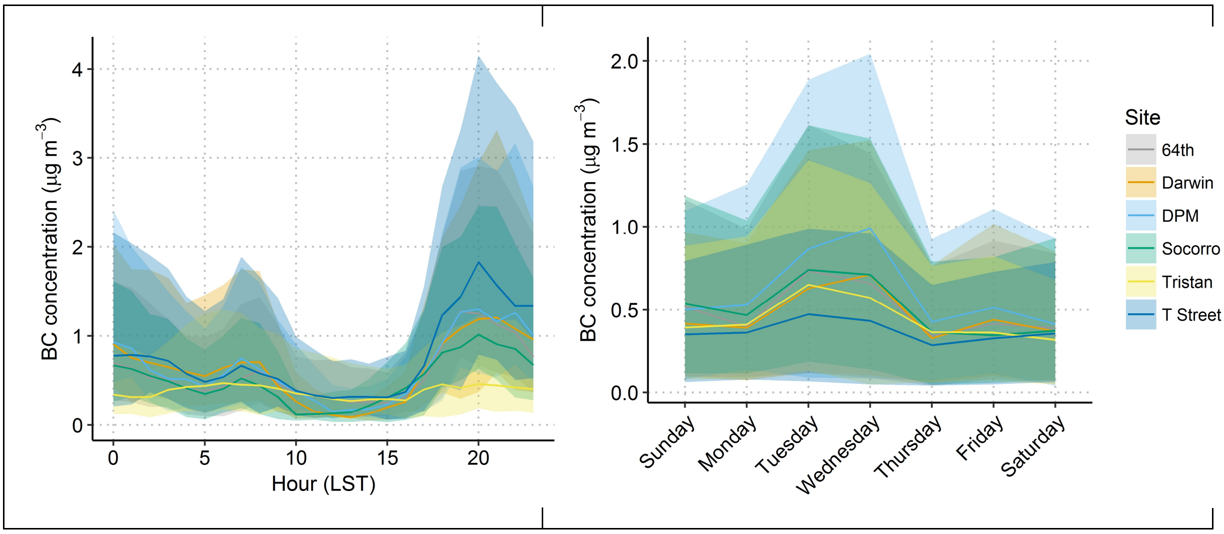

Figure S1. Diurnal (left) and day-of-week (right) plots for BC by site; the median is shown as a line, and the shading indicates the 95th confidence interval around the median. The 95th confidence interval is calculated based on the rank ordered data values obtained from the binomial quantile function at probabilities 0.025 and 0.975. DPM is Del Paso Manor.

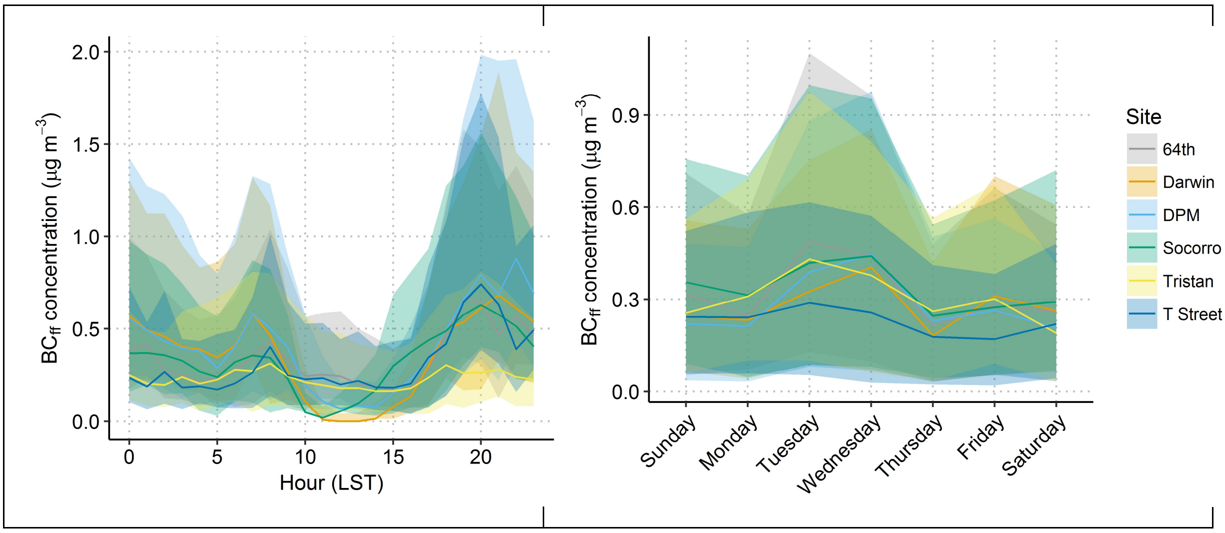

Figure S2. Diurnal (left) and day-of-week (right) plots for BC_{ff} by site; the median is shown as a line, and the shading indicates the 95th confidence interval around the median. The 95th confidence interval is calculated based on the rank ordered data values obtained from the binomial quantile function at probabilities 0.025 and 0.975. DPM is Del Paso Manor.

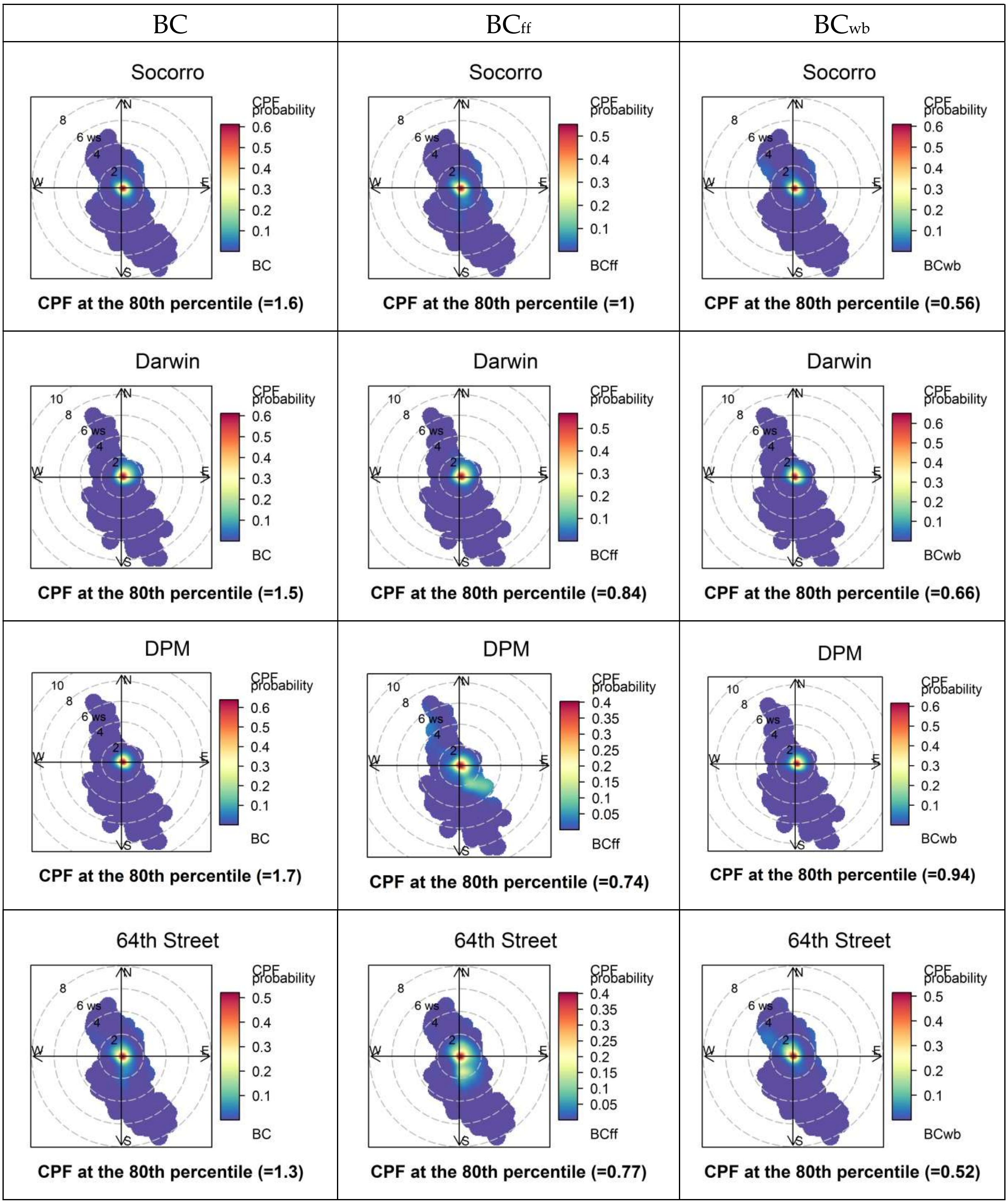

(Figure continued on following page.)

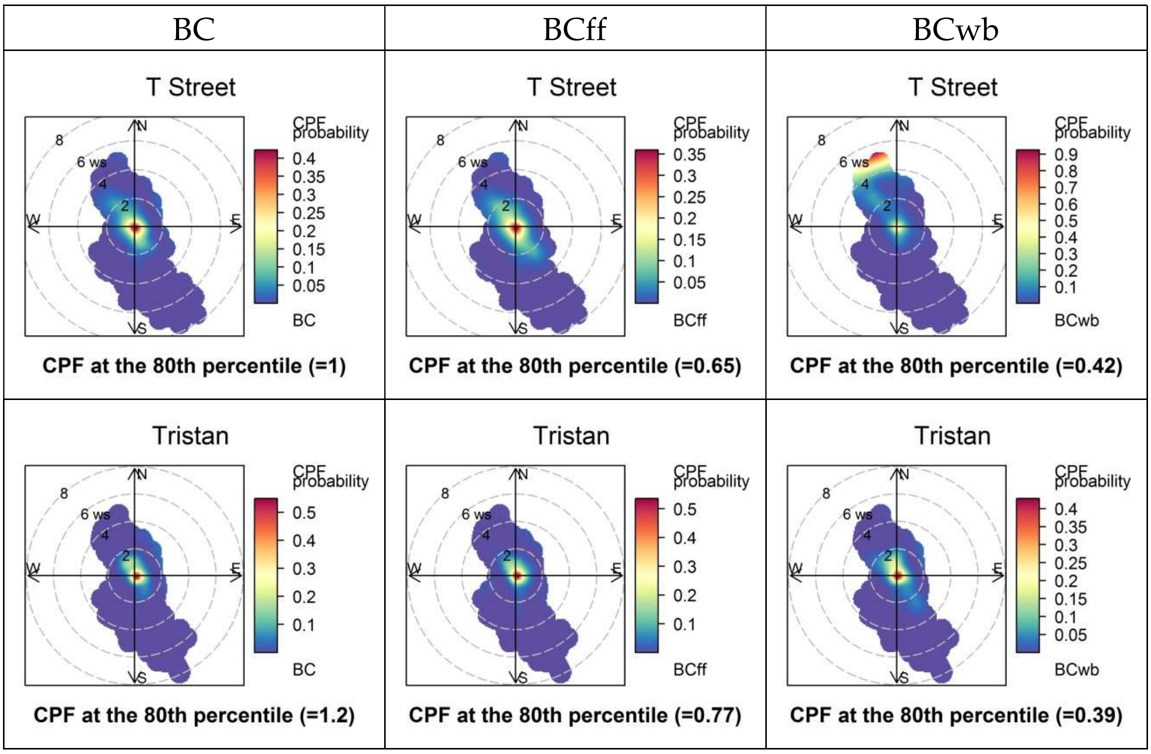

Figure S3. CBPF plots for the highest quintile of BC (left column), BC_{ff} (middle column), and BC_{wb} (right column) concentrations, labeled by site. Color indicates the probability that the pollutant concentrations measured at a given wind speed and direction fell within the top quintile of all pollutant concentrations measured at that site (the CPF probability). Wind speeds are denoted in the concentric circles (m/s). Red and yellow indicate that concentrations within the top quintile are more likely.

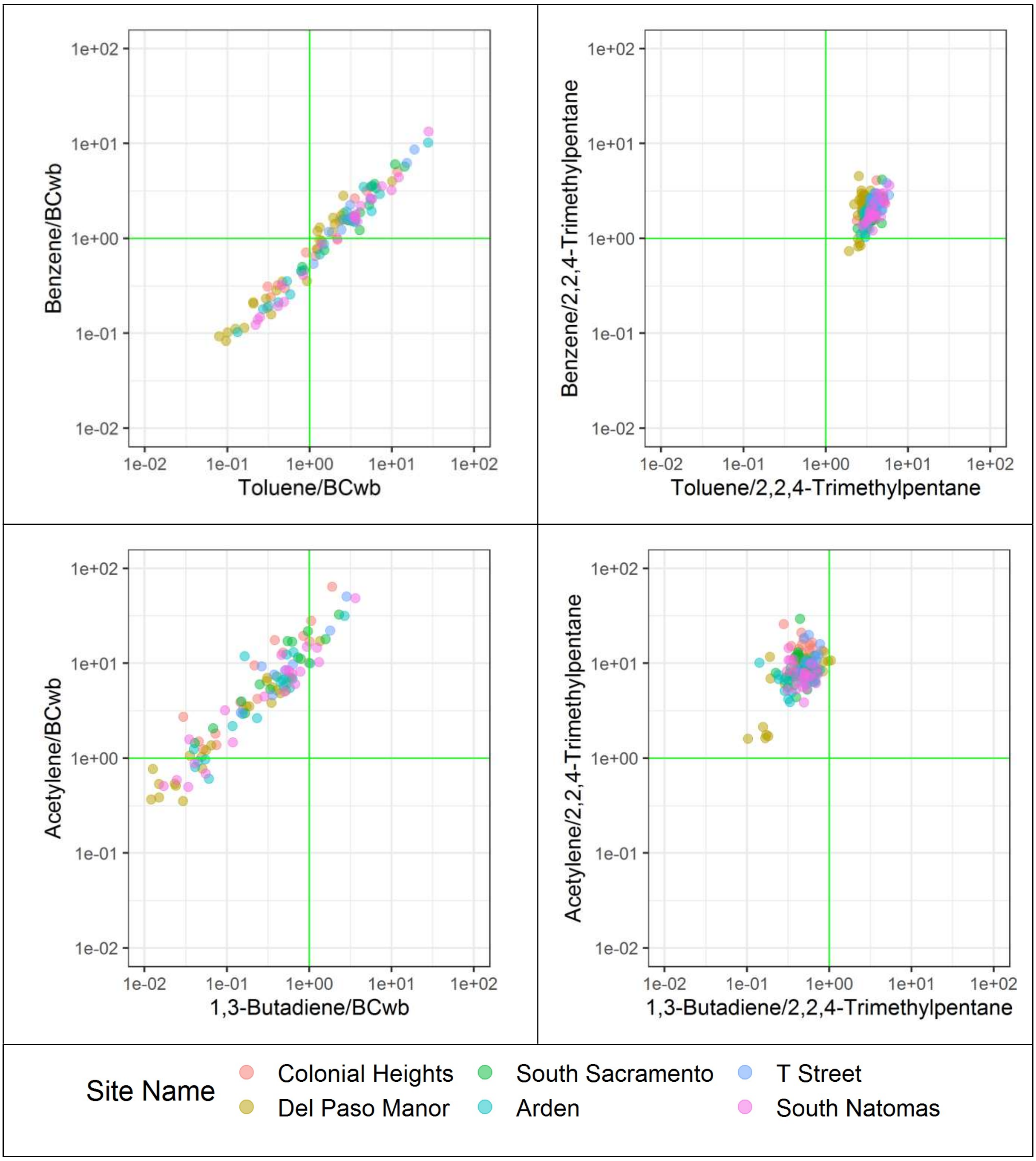

Figure S4. Ratio:ratio plots of (top) benzene and toluene and (bottom) acetylene and 1,3-butadiene, divided by BC_{wb} (left) and 2,2,4-trimethylpentane (right).

Questionnaire Details

METHODOLOGY:

Field Dates: • Mid December 2016 and/or Early Jan 2017
Sample Size: • 900 completed interviews (450 cell and 450 landlines)
Sampling Error (calculated at 95% confidence level): • +/- 3.3% for 900
Unit of Analysis: • Head of Households in Sacramento County in which an indoor (or outdoor) wood (or pellet) burning device exists
Language: • English, Spanish, and Russian
Sampling Frame: • RDD purchased sample, proportionally representative of population
Quotas: • Gender 50/50
Average Length of Interview: • 8 minutes

• *INTRODUCTION* •

Hello, my name is _______________ from Meta Research, a public opinion research firm. We are conducting a survey about air quality issues facing our local area. This is not a solicitation and you will not be asked to buy anything.
May I speak to the someone in your household who is presently at home and is considered to be a head of household?
[If head of household] Can you take time now for a confidential interview?
[When speaking with head of household] Hello, my name is _______________ from Meta Research, a public opinion research firm. We are conducting a survey about air quality issues facing our local area. This is not a solicitation and you will not be asked to buy anything. Can you take time now for a confidential interview?
[IF NO HEAD OF HOUSEHOLD AVAILABLE, SCHEDULE CALL BACK TIME]
[IF NECESSARY: It should take approximately 8 minutes, depending on your responses.]
[IF NECESSARY: Everything you tell me will be completely confidential. You have the right to refuse to answer any question at any time. I can conduct the interview right now, or we can make an appointment for me to call you back at a more convenient time.]
[IF NECESSARY: We can share the name of sponsor at the end of the survey so as not to bias your responses.]
[IF NECESSARY: This is a research study and I'm only interested in your opinions as a Sacramento area resident. At no time will I try to sell you anything.]

• *DATABASE INFORMATION* •

DB1. ZIP Code (FROM SAMPLE):

• *SCREENING QUESTIONS* •

READ TO ALL
Thank you. This call may be monitored for quality control purposes.
S1. To confirm, can you tell me your zip code?
[RECORD RESPONSE]
[ASK ALL RESPONDENTS]
S2. And, do you have a wood-burning device in or outside your home; such as a fireplace, a wood or pellet stove; or an outdoor fire?

[NOTE TO INTERVIEWER: WE ARE LOOKING TO SPEAK WITH THOSE WHO HAVE THE CAPABILITY TO BURN WOOD OR PELLETS. INDOOR GAS UNITS AND OUTDOOR BARBEQUES OF ANY KIND DO NOT COUNT]

1. Yes (continue)
2. Yes but just a barbeque (volunteered) (THANK & TERMINATE)
3. No (THANK & TERMINATE)

8) Don't know (THANK & TERMINATE)
9) Refused (THANK & TERMINATE)

[ASK IF S2 =1]

S3. And what wood-burning device or devices do you have? (Interviewer record all that apply: multi-punch)

1) Indoor fireplace (burns wood, pellets, or logs – NOT gas)
2) Fireplace insert
3) Wood or pellet stove
4) Outdoor wood burning fire pit
5) Chiminea [pronounced chee-men-A-uh] [Thank and Terminate if this is the ONLY device]
6) Outdoor Barbeque: [Thank and Terminate if this is the ONLY device]
8) Don't know (VOLUNTEER)
9) Refused

[ASK IF S3 =1]

S3. Is burning wood or pellets the only possible way to heat your home or can you heat it with another permanent heat source?

1) Wood-burning is the only heat source [THANK & TERMINATE]
2) Other sources available to heat home [CONTINUE]
8) Don't know / Undecided [VOLUNTEERED] [THANK & TERMINATE]
9) Refused [VOLUNTEERED] [THANK & TERMINATE]

[ALL RESPONDENTS]

S4. [BY OBSERVATION] Gender [QUOTAS: 50/50 SPLIT]

1) Female
2) Male

[ALL RESPONDENTS]

S5. [BY OBSERVATION] Language

1) English
2) Spanish
3) Russian

• *SURVEY BEGINS* •

• *AIR QUALITY ISSUES* •

[ASK ALL RESPONDENTS]

1.1 Now, I'd like to talk about air quality issues in the Sacramento area. Using the scale, not at all [1], somewhat [2], or very unhealthy [3], how would you rate the contribution to WINTERTIME air pollution caused by ___________________ in the Sacramento area?

[IF NECESSARY: And by winter, I mean from November through February? [FOR NEXT: And how would you rate the seriousness of WINTERTIME air pollution caused by ____?]

[CATEGORIES FOR CODING]

1) Not at all unhealthy
2) Somewhat unhealthy
3) Very unhealthy
8) Undecided/Don't know [VOLUNTEERED]
9) Refused

RANDOMIZE
a. traffic
b. industry
c. agricultural burning
d. residential wood burning fireplaces

• WOOD BURNING ACTIVITY •

“Now let’s talk about your wood burning activity this winter, that is, from late November to today.”
[ASK IF S3 = 1]
4.0a In general, would you say you burned wood, pellets, or manufactured logs in your indoor fireplace ____ [READ LIST
1) Less than once a week
2) About once a week
3) Two or more times a week
4) Mainly on Friday and Saturday nights
5) Mainly on Holidays
6) Not at all
8) Other
9) Don’t know/Undecided [VOLUNTEERED]
10) Refused [VOLUNTEERED]
[ASK IF S3 = 1 & 4.0a = 1, 2, 3, 4, 5, or 8]
4.0b And typically, what time of the day do you burn with your indoor fireplace? Day, night, or both?
1) Day (6 am to 6 pm)
2) Night (6 pm to 6am)
3) Day and night
8) Other [RECORD RESPONSE]
9) Don’t know/Undecided [VOLUNTEERED]
10) Refused [VOLUNTEERED]
[ASK IF S3 = 1 & 4.0a = 1, 2, 3, 4, 5, or 8]
4.0c Do you have an Environmental Protection Agency certified indoor fireplace?
1) It is certified
2) Not certified
8) Don’t know [VOLUNTEERED]
9) Refusal [VOLUNTEERED]
[ASK IF S3 = 2]
4.1a In general, would you say you burned wood, pellets, or manufactured logs in your fireplace insert ____ [READ LIST
1) Less than once a week
2) About once a week
3) Two or more times a week
4) Mainly on Friday and Saturday nights
5) Mainly on Holidays
6) Not at all
8) Other
9) Don’t know/Undecided [VOLUNTEERED]
10) Refused [VOLUNTEERED]
[ASK IF S3 = 2 & 4.1a = 1, 2, 3, 4, or 5]
4.1b And typically, what time of the day do you burn with your fireplace insert? Day, night, or both?
1) Day (6 am to 6 pm)
2) Night (6 pm to 6am)
3) Day and night

8) Other [RECORD RESPONSE]
9) Don't know/Undecided [VOLUNTEERED]
10) Refused [VOLUNTEERED]

[ASK IF S3 = 2 & 4.1a = 1, 2, 3, 4, 5, or 8]
4.1c Do you have an Environmental Protection Agency certified fireplace insert?

1) It is certified
2) Not certified
8) Don't know [VOLUNTEERED]
9) Refusal [VOLUNTEERED]

[ASK IF S3 = 3]
4.2a In general, would you say you burned wood, pellets, or manufactured logs in your wood or pellet stove ____ [READ LIST

1) Less than once a week
2) About once a week
3) Two or more times a week
4) Mainly on Friday and Saturday nights
5) Mainly on Holidays
6) Not at all
8) Other
9) Don't know/Undecided [VOLUNTEERED]
10) Refused [VOLUNTEERED]

[ASK IF S3 = 3 & 4.2a = 1, 2, 3, 4, or 5]
4.2b And typically, what time of the day do you burn with your wood or pellet stove? Day, night, or both?

1) Day (6 am to 6 pm)
2) Night (6 pm to 6am)
3) Day and night
8) Other [RECORD RESPONSE]
9) Don't know/Undecided [VOLUNTEERED]
10) Refused [VOLUNTEERED]

[ASK IF S3 = 3 & 4.2a = 1, 2, 3, 4, 5, or 8]
4.2c Do you have an Environmental Protection Agency certified wood or pellet stove?

1) It is certified
2) Not certified
8) Don't know [VOLUNTEERED]
9) Refusal [VOLUNTEERED]

[ASK ALL RESPONDENTS]
4.1 And in general would you say you burned less, the same, or more wood, pellets or manufactured logs this past winter as compared with a typical winter?
[Interviewer: This includes both indoor and outdoor devices]

1) Less
2) Same
3) More
8) Don't know/Undecided [VOLUNTEERED]
9) Refused [VOLUNTEERED

4.3 And in general, where did you get the wood, pellets, or manufactured logs you've burned this winter?

1) Grocery Store
2) Hardware Store
3) Wood Dealer
4) Got it free
8) Other

9) Don't know/Undecided [VOLUNTEERED]
10) Refused [VOLUNTEERED]

• *AWARENESS OF PM POLLUTION& NO BURN* •

[ASK ALL RESPONDENTS]
5.0 This winter, did you hear, read, or see anything informing residents not to use their wood burning fireplaces or outdoor fire pits because of poor air quality?
[IF NECESSARY: Again by winter, I'm talking about late November.]
1) Yes
2) No
8) Don't know/Undecided [VOLUNTEERED]
9) Refused [VOLUNTEERED]
[ASK IF 5.0=1]
5.1 And where did you read, see or hear this information?
CATEGORIES FOR CODING
1) Facebook
2) Twitter
3) Website
4) Mobile app
5) Air Alert email
6) Phone recording
7) Newspaper
8) Television
9) Radio
10) Word of Mouth
11) Other (Specify)
12) Don't Know
13) Refused

[ASK IF 5.0=1]
5.2 And did you reduce the number of times you burned this winter because you heard or saw a notice not to burn wood?
[IF NECESSARY: Again by winter, I'm talking since the beginning of December.]
1) Yes
2) No
3) Yes – received an Air Alert [VOLUNTEERED]
4) Never burned
8) Don't know/Undecided [VOLUNTEERED]
9) Refused [VOLUNTEERED]
[READ if 5.0 = 2, 8 or 9]
You may or may not have heard that in Sacramento County, it's the law that from November through February residents and businesses are prohibited from using indoor or outdoor fireplaces, wood stoves, and fire pits that burn wood, pellets, manufactured logs or any other solid fuel on days when air quality is forecast to be unhealthy to breathe. It is your responsibility to Check Before You Burn, to see if it is permissible to light a fire.
[ASK IF 5.0 = 2, 8 or 9]
5.3 Does this now sound familiar to you?
CATEGORIES FOR CODING:
1) Yes
2) No
8) Undecided/Don't know [VOLUNTEERED]

9) Refusal [VOLUNTEERED]

[ASK IF 5.3 = 1]

5.4 And did you reduce the number of fires you burned this winter because you heard or saw a notice not to burn wood?

[IF NECESSARY: Again by winter, I'm talking since the beginning of December.]

1) Yes
2) No
3) Yes – received an Air Alert [VOLUNTEERED]
4) Never burned
8) Don't know/Undecided [VOLUNTEERED]
9) Refused [VOLUNTEERED]

DEVICE CHARACTERISTICS

[ASK ALL RESPONDENTS]

6.0 Are you aware that you can apply for a sole source of heat or economic hardship waiver to be considered exempt from burn bans if you are approved by the Air District?

1) Yes
2) No
3) Yes, already exempt (Volunteered)
8) Don't Know/Undecided (Volunteered)
9) Refused (Volunteered)

[ASK ALL RESPONDENTS]

7.0 Are you aware of the complaint line to call to alert the Sacramento Air District if you see someone burning on a day when burning is prohibited?

1) Yes
2) No

• *DEMOGRAPHICS* •

"Now just a few final questions for statistical purposes… "

[ASK ALL RESPONDENTS]

8.0 How many people are living in your household?

1) Live alone
2) 2
3) 3
4) 4
5) 5 or more
9) Non-response (Don't know/Refused)

[ASK ALL RESPONDENTS]

8.1 Please stop me when I reach the category that includes your age.

[READ CHOICES]

1) 18 – 24
2) 25 – 34
3) 35 – 44
4) 45 – 54
5) 55 – 64
6) 65 & older
9) Non-response (Don't know/Refused)

[ASK ALL RESPONDENTS]

8.2 What is the highest level of education you have completed?

[IF NECESSARY: READ CHOICES]

1) High school or less
2) Some college
3) Trade or vocational school – no college
4) College degree
5) Post graduate degree
9) Non-response (Don't know/Refused)

[ASK ALL RESPONDENTS]

8.3 Please stop me when I reach the category that best describes your ethnic background. [NOTE: ORDER IS ALPHABETICAL]

1) African-American
2) Asian/Pacific Islander
3) Caucasian
4) Hispanic/Latino
5) Russian
6) Something else [CAPTURE RESPONSE]
9) Non-response (Don't know/Refused)

[ASK ALL RESPONDENTS]

8.4 And finally, please stop me when I read the category that best describes your TOTAL household income before taxes for 2013.

1) Under $20,000
2) $20,000 to less than $40,000
3) $40,000 to less than $60,000
4) $60,000 or to less than $100,000
5) $100,000 or more
9) Non-response (Don't know/Refused)

© 2019 by the authors. Submitted for possible open access publication under the terms and conditions of the Creative Commons Attribution (CC BY) license (http://creativecommons.org/licenses/by/4.0/).
